# Manipulation of Developing Juvenile Structures in Purple Sea Urchins (*Strongylocentrotus purpuratus*) by Morpholino Injection into Late Stage Larvae

**DOI:** 10.1371/journal.pone.0113866

**Published:** 2014-12-01

**Authors:** Andreas Heyland, Jason Hodin, Cory Bishop

**Affiliations:** 1 Integrative Biology, University of Guelph, Guelph, Ontario, Canada; 2 Hopkins Marine Station, Stanford University, Pacific Grove, CA, United States of America; 3 Department of Biology, St. Francis Xavier University, Antigonish, NS, Canada; Chang Gung University, Taiwan

## Abstract

Sea urchins have been used as experimental organisms for developmental biology for over a century. Yet, as is the case for many other marine invertebrates, understanding the development of the juveniles and adults has lagged far behind that of their embryos and larvae. The reasons for this are, in large part, due to the difficulty of experimentally manipulating juvenile development. Here we develop and validate a technique for injecting compounds into juvenile rudiments of the purple sea urchin, *Strongylocentrotus purpuratus*. We first document the distribution of rhodaminated dextran injected into different compartments of the juvenile rudiment of sea urchin larvae. Then, to test the potential of this technique to manipulate development, we injected Vivo-Morpholinos (vMOs) designed to knock down p58b and p16, two proteins involved in the elongation of *S. purpuratus* larval skeleton. Rudiments injected with these vMOs showed a delay in the growth of some juvenile skeletal elements relative to controls. These data provide the first evidence that vMOs, which are designed to cross cell membranes, can be used to transiently manipulate gene function in later developmental stages in sea urchins. We therefore propose that injection of vMOs into juvenile rudiments, as shown here, is a viable approach to testing hypotheses about gene function during development, including metamorphosis.

## Introduction

Sea urchins have been a favored study organism in fields ranging from ecology to molecular biology for over 100 years. They are abundant and diverse from the poles to the equator, and from the deep sea to the intertidal. There is a rich fossil record dating back about 450 million years to the late Ordovician [1], and sea urchins have many unique body plan features and adaptations, including pentameral symmetry, a water vascular system, mutable collagen [2],[3] and larval budding and regeneration [4]–[6]. Finally, there is a global fishery worth almost half a billion US dollars [7] which motivates a deeper understanding of sea urchin biology.

Sea urchins have long been a preferred laboratory organism due to the ease of obtaining large quantities of gametes, which can be fertilized externally and used in studies of fertilization, embryogenesis and larval development [8]–[12]. It was primarily for these latter reasons that the genome of the purple sea urchin *Strongylocentrotus purpuratus* was the first free-living non-chordate marine invertebrate to be sequenced [13]. The advent of urchin genomics has heralded renewed interest in urchin development, and paired with modern manipulation techniques such as morpholino microinjection [14],[15], the sea urchin is now one of only a handful of animals whose embryos are readily amenable to both classical and contemporary embryological techniques, including blastomere separations, cell transplantation, and more recently, genetic manipulations [10],[16]–[20]. While remarkable progress has been made in understanding the molecular and cellular basis of development in sea urchin embryos, comparatively little is known about the development of the adult body plan as the planktonic larva transitions to the benthic juvenile. That is, the range of experimental approaches enjoyed by urchin embryologists has not been applied to the development of juvenile tissues.

Complex life histories –development through feeding planktonic larvae and metamorphosis to benthic juveniles– are widespread in the ocean, with numerous hypothesized independent origins of complex from simpler ancestral life cycles [21]–[23]. Even more numerous are losses of larval feeding and/or planktonic development hypothesized for many metamorphic phyla [24]–[27]. Thus, despite the importance and commonality of complex life cycles in marine organisms, we have little understanding of the internal and environmental factors that regulate the progression of such life cycles in even a single marine species.

Sea urchins display one of the most dramatic metamorphic transitions among the animals. Their larvae are bilaterally symmetric, but their juveniles begin development as an asymmetric invagination of larval epithelial cells, which then, in concert with coelomic tissues undergo morphogenesis into a juvenile rudiment, all internal to the larval epithelium [28]–[30]. During juvenile rudiment development, the pentameral symmetry of the adult forms along with the primordia of many juvenile structures. After larvae having well-developed juvenile rudiments settle to the sea floor and select an appropriate benthic substrate, they rapidly undergo the most dramatic stage of the metamorphic transformation: in a matter of minutes the juvenile everts out of the larval body, the larval ectoderm I s withdrawn, and the juvenile begins to move along the sea floor using its tube feet [31].

While this life cycle transformation in sea urchins has fascinated biologists for centuries, detailed functional studies of late larval development and the metamorphic transition have been lacking, due in large part to lengthy larval periods and the inherent limitations of accessing densely packed forming juvenile tissue. Still, indirectly developing sea urchin larvae are an ideal organism with which to gain insight into juvenile morphogenesis and metamorphosis. With proper technique, large numbers of sea urchin larvae can be reared synchronously to metamorphic competence [11], detailed descriptions of metamorphic stages have been published [28],[30],[32] and many transient knockdown techniques have been applied to sea urchin embryos [14]–[20].

Yet one significant challenge remains: how does one experimentally manipulate the development of juvenile tissues in sea urchins? In the taxa for which we have the greatest understanding of mechanisms of metamorphosis -insects- it took many technical advancements (such as heat shock and Flp-recombinase technology) to begin to illuminate metamorphic development. The main reason for this difficulty is that standard genetic manipulations -whether through stable mutagenesis or through factors injected in the egg- generally lead to embryonic phenotypes or lethality, and their larvae would thus be abnormal or not survive to the metamorphic stages under study. More general treatments of larvae likewise cause larval phenotypes independent of their impacts on the structures in larvae fated to form the juvenile: namely, the imaginal discs in holometabolous insects. Also, years of detailed studies in insects led to the ability to culture these imaginal disks *in vitro* (and *in vivo* through their injection into host larvae), thus illuminating the previously obscure events of specification and differentiation of presumptive adult structures. We are not aware of any such techniques having been devised for a marine invertebrate, though new functional genomics approaches –that allow the specific manipulation of genes and gene products– should allow more incisive investigations into marine invertebrate larval development, including settlement and metamorphosis.

Here we report on our success in targeted manipulation of juvenile development in purple sea urchin larvae. Using a new class of morpholino oligonucleotides that readily cross cell membranes (Vivo-Morpholinos - vMOs), we describe a technique where we can reliably inject these compounds inside juvenile rudiment tissues of temporarily immobilized late stage *S. purpuratus* larvae. As a proof of concept, we injected vMOs designed to knock down the expression of p16 and p58b, two genes involved in skeletal elongation in sea urchin embryos [33],[34]. We report a significant decrease in the elongation rate of adult spines in both p16 and p58b-injected larvae when compared to those injected with the control vMO, a carrier control and an uninjected control.

Along with our morpholino injections, we also injected rhodaminated dextran (RD) as a tracer. These injections resulted in accumulation of rhodamine into different compartments of the developing rudiment, allowing for visualizations of developing juvenile tissues at a level of detail not previously described.

## Materials and Methods

We conducted all experimental work and culturing of embryos and larvae in the summer of 2013 and 2014 in either the laboratory of Dr. Christopher Lowe at Hopkins Marine Station of Stanford University (HMS in Pacific Grove CA, USA) using UV-treated, 0.45 µm Milipore-filtered natural sea water (MFSW), or in A.H.'s laboratory at The University of Guelph (Guelph ON, Canada) using 0.2 µm Milipore-filtered (Instant Ocean) artificial sea water (MFASW).

### Adult urchins

All sea urchins were originally obtained from The Cultured Abalone Ltd (Goleta CA, USA). At HMS, urchins are maintained in flow through natural seawater tanks at ambient temperatures (usually between 10°C and 14°C) and an average salinity of 34ppt, and fed fresh kelp blades (mostly *Macrocystis pyrifera*) *ad libitum* year round. In Guelph, urchins are maintained in artificial seawater (Instant Ocean) at 12°C and 34ppt salinity, and fed rehydrated kombu kelp (*Laminaria sp.*) *ad libitum* year round.

### Larval culturing

We spawned male and female sea urchins by gentle shaking or intra-coelomic injection with 0.5 M KCl, and fertilized spawned eggs (>90% fertilization success) with diluted sperm using standard methods [11]. We set up each of six fertilizations in 2013–2014 (16 June, 25 June and 3 July 2013 at HMS; 4 and 20 September 2013 and 12 June 2014 in Guelph) using three females and one male, and then mixing approximately equal numbers of fertilized eggs from all three crosses. In Guelph, we only cultured embryos for 72 hours for the embryo soaking experiments described below; we conducted all larval culturing for the injection experiments at HMS.

Starting on day 3 after fertilization, we fed larvae a mixture of *Dunieliella tertiolecta* (3 cells/µl) and *Rhodomonas spp.* (2.5 cells/µl) every 2 days following water changes by reverse-filtration of >95% of the culture volume. Initial larval densities were approximately 1 larva/ml. On day 14, we reduced the density to approximately 1 larva/6 ml. We monitored developmental progression of larvae using a new standardized staging scheme based upon rudiment soft tissue and skeletal elements [28], using a compound microscope (Zeiss AxioImager Z1 with AxioCam Mrm and MRc5 Cameras) equipped with cross-polarized light. We attempted injections at various stages starting at approximately 21 days after fertilization, before the initial appearance of juvenile skeletal elements [28]. While we were able to successfully inject larvae from this stage through at least skeletogenic Stage 8 [28], the results presented here on experimentally manipulated skeleton elongation were all obtained from larvae that we injected at the stage when incipient spines ("pre-spines") are visible (skeletogenic Stage 7, see below and [28]).

### Validation of Vivo-Morpholino (vMO) oligonucleotides

vMOs are designed to enter the cell by crossing the plasma membrane without any additional modification necessary. This has been shown for cultured cells and through intravenous injection in mammals [35]. Moreover, recently published data shows that sea urchin embryos and larvae soaked in vMOs show clear knockdown phenotypes for BMP signaling [36]. Because the particular vMOs we used in this study had not been validated in sea urchin embryos and larvae via soaking experiments, we assessed the capacity of p58b vMO to generate expected embryonic phenotypes. First we incubated post-gastrula *S. purpuratus* embryos in MFASW containing p58b or p16 vMO and compared the resulting phenotype to published data on injections of a standard p58b or p16 MO [33],[34]. Second we used RT-PCR to test for a reduction of the properly spliced p58b form in exposed embryos compared to controls. Table 1 summarizes the vMO sequences used (identical to the sequences used for the standard MO in [33],[34]

**Table 1:** Vivo-Morpholino sequences used in the present study.

Target	Type	Sequence	Reference
p58b	Splice site blocking	5′-ACGGCTTCCATCACTAACCTGATTG-3′	Adomako-Ankomah and Ettensohn 2011 [33]
p16	Translation blocking	5′-GGTCTTCATAGTAATAGTGTGTGTA-3′	Cheers and Ettehsohn 2005 [34]
Control	Control	5′-CCTCTTACCTCAGTTACAATTTATA-3′	GeneTools LLC

References listed indicate studies that have confirmed the effectiveness of these MO sequences. Note that no p58b or p16 sequences have ever been tested with Vivo-Morpholinos.

We began our MO soaking treatments in post-gastrula stage embryos because when we treated newly-fertilized embryos with control, p58b or p16 vMOs at 15 µM, we observed an irreversible inhibitory effect on gastrulation (data not shown), indicating a non-specific effect of early embryonic exposure to this concentration of vMOs. Notably, skeletogenesis proceeded independently of this inhibitory effect in the control vMO treatment, but was inhibited by p58b and p16 vMO treatment (data not shown). Nevertheless, to circumvent the non-specific gastrulation defect, we report only on vMO exposures beginning in late gastrulae.

For these soaking experiments, we cultured embryos for 48 hours at 14°C until triradiate spicules began to form. Then we transferred 400 embryos per well of a 24 well plate (Corning Life Sciences C353095) containing 500 µl of MFASW and either 5, 10 or 15 µM p58b or p16 vMO, control vMO or milliQ water (as a carrier control, since we used milliQ water to dilute the vMO stock solutions). After a 48 h exposure at 14°C, we fixed a subset of embryos with 4% formaldehyde (dissolved in milliQ water from 16% ultra-pure stock solution) and immediately visualized them on a Nikon Ti inverted microscope equipped with cross-polarized light to examine growth of larval skeletal rods. We analyzed ten embryos per treatment using ImageJ v 1.47. We computed a skeletal length for each embryo by summing the lengths of both the left and right postoral rods, and then compared the mean total postoral rod lengths among treatments using a one-way ANOVA in SPSS (v21).

For RT-PCR, we used the remainder of the 48 h-exposed embryos described above and fixed them in 300 µl TRIZOL. We extracted total RNA using previously published protocols [37], and then synthesized cDNA using the MultiScribe High Capacity cDNA Reverse Transcription Kit (Applied Biosystems) following the manufacturer's instructions. We diluted cDNA 1∶50 and used it as a template for PCR using BioRad iProof High Fidelity DNA polymerase (BioRad - 172-5301) following manufacturer's instructions. The T_m_ for PCR was 56°C and primers used were as follows:

- p58bF: CATGCTGGAGAGTTCATTGGGTTCGC
- p58bR: GCACTCTAGACTGTCATTGGGTCCGT.

After 35 cycles we analyzed the results of the PCR experiment on an agarose gel using RedSafe Nucleic Acid Staining Solution (iNtRON Biotechnology, Inc.) and imaged on a red imaging system (Cell Biosciences).

### Expression of target genes in larval and juvenile tissues

We quantified skeletal gene expression in larval and juvenile tissues by subjecting RNA extracted from different stages to qRT-PCR. We first used RNAqueous micro kit (Ambion) to extract RNA from three different developmental stages: 2-arm larvae with only larval skeleton (day 4), competent larvae with rapidly forming juvenile skeleton (day 43), and juveniles 12 hours after settlement [38] with no larval arms or skeleton remaining. We synthesized cDNA using AB systems cDNA synthesis kit. For the qRT-PCR reaction, we used PerfeCTa SYBR Green FastMix (Quanta) with Tm set to 59°C. We pooled two independent biological samples and generated three technical replicates for qRT-PCR. We used the following primers (from [39]):

- Sp-p16 F: CAATTCAATTTCGGCAACAC
- Sp-p16 R: CCTCCAAGACCATCCAGACT
- Sp-p58B F: CTGAACGAAGCACAGTCGAT
- Sp-p58B R: CTGCATGTCCTTTGGAACAC
- Sp-Ubq F: CACAGGCAAGACCATCACAC
- Sp-Ubq R: GAGAGAGTGCGACCATCCTC

### The injection protocol and validation of microinjection technique

We conducted several rounds of preliminary injections with rhodaminated dextran (RD). Our objectives were to (i) refine the technique itself, including needle size, injection pressure and injection volume, (ii) establish that a visual assessment of the position of the needle in the rudiment could be expected to correspond to the deposition of dye in a particular region of the rudiment, (iii) assess the feasibility of injecting into larvae having rudiments at different stages of development, (iv) assess the retention of dye injected into rudiments over time, as it related to their stage of development and the original site of injection and (v) assess the survivorship of injected larvae. We injected into rudiments using 0.04% RD using injection conditions described below. In preliminary experiments we injected 137 rudiments in seven separate trials.

We prepared needles for injection using Sutter Instrument thin wall borosilicate tubing with filament (Sutter Instruments; #BF100-78-100, O.D. 1.0 mm; I.D. 0.78 mm; fire polished) pulled on a Sutter (Model P-87) needle puller using a program with the following parameters: Heat 010; Pull 110; Velocity 75; Time 200; Pressure 300. After breaking the needle tip in mineral oil, we adjusted the pressure in order to produce an air droplet with approximate diameter of 250 µm, estimated by comparison to a preset mark carved into a glass slide, and then loaded the needle with the required injection reagent.

We injected larvae using a Zeiss Primo Vert equipped with 4x, 10x, 20x, and 40x objectives and 10x ocular magnification (i.e., 40−400x total magnification). The injection apparatus consisted of: (i) two micromanipulators (Applied Scientific Instruments) raised a few centimeters above the microscope stage and held in place with retort stands– one micromanipulator held the injection needle, the other held the suction pipette; (ii) a suction pipette made from a 10 ml syringe connected to an extra-long gel-loading 10 µl plastic micropipette tip (Eppendorf micro loader 100 mm). Note that the base of the micropipette tip was fitted to the tip of 10 ml syringe to accomplish an airtight fit; and (iii) an injection needle attached to the injector (MPPI-3 Milli-Pulse Pressure Injector).

For each injection, we placed one larva in a 35 mm diameter polystyrene Petri dish filled with MFSW that was previously cooled to 14°C. Petri dishes were modified by cutting holes in the center and then covering the hole with cover glass; this improved the optics allowing for higher magnification examination. At 40x magnification, we manually oriented the larva so that the right posterior side of the larva was facing the micropipette tip of the suction pipette (i.e., with the juvenile rudiment facing away from the micropipette tip). We then aspirated the larva against the micropipette tip by gently applying suction. In no cases did this technique rupture the larval epithelium.

Once the larva was immobilized, we placed the injection needle at an approximately 40° angle above the rudiment and inserted it through the larva epithelium. Then, we brought the needle closer to the juvenile rudiment under 100x and 200x magnification and finally pushed it through the vestibular ectoderm or further into other compartments of the rudiment. The exact location of injection varied in preliminary rounds of injections and in dye tracing experiments. To assess the fate of injected compounds, we also estimated dye localization after 48 and 96 hours (not shown).

We assessed the results of all preliminary injection trials (i.e., for validating the injection technique) in live larvae, with the exception of one trial, in which we fixed larvae at various times post-injection in 4% formaldehyde prepared in MFSW and then stained them with DAPI. We visualized live larvae using epi-fluorescent illumination on a Zeiss (Zeiss AxioImager Z1 equipped with AxioCam Mrm and MRc5 cameras) compound microscope equipped with appropriate filters, and DAPI stained larvae on a Zeiss LSM710 confocal microscope. Image stacks were projected from Zeiss.czi image files using Image J (v10.2) with the LOCI Tools plug-in (http://downloads.openmicroscopy.org/bio-formats/4.4.10/). We constructed figures with Adobe Photoshop CS5 (v12.1) and Deneba Canvas (vX).

### Microinjection of Vivo-Morpholino oligonucleotides (vMOs) and experimental design

We present injection data using vMOs [GeneTools (LLM)] for two genes that function in larval skeletal elongation, p58b and p16 [33],[34], as well as a vMO control, a rhodaminated dextran (RD) control, and an uninjected control. We loaded and calibrated injection needles as described above, with 20 µM vMO in 0.04% RD, or 0.04% RD alone.

Prior to the injection experiments reported here, we first staged larvae by mounting them individually on slides with raised cover glass and examined them microscopically. We only used Stage 7 larvae for the injections, which were those having spines at the "pre-spine" stage (see below and [28]). All mounted larvae were immobilized under cover glass for fewer than 15 minutes, and aside from the 2 minutes maximum that each slide was on the microscope stage, we maintained the slides at 14°C on a cooling plate. After initial staging, we gently transferred Stage 7 larvae to individual wells in a 24 well plate (Falcon 351147 polystyrene non-tissue culture treated, flat bottom, low evaporation lid) at 14°C for a>1 hour recovery before injection. Each experimental trial consisted of four larvae per treatment (p58b vMO, p16 vMO, uninjected control, vMO control, RD control) and we performed a total of six trials. This resulted in twenty-four replicate larvae for each treatment.

After injection, we withdrew the needle and released the larva from the suction pipette and transferred it into a recovery well of a 24 well plate containing 1.5 ml of MFSW at 14°C, with food at the same concentrations as used throughout larval culturing (see above). The average time to manipulate and inject larvae was approximately 10 minutes. Every 15–20 minutes, we exchanged a portion of the water in the injection dish with MFSW cooled to 14°C.

### Experimental analysis

After injection, we cultured each larva for 96 hours in individual wells of a 24 well plate, with a full water change and new food after 48 hours. Then, at 96 hours, we scored each larva in a single-blind fashion: the person scoring the phenotype did not know the treatment to which the larva had been subjected, while the persons mounting larvae and recording data did. First, we mounted the larva with raised cover glass, categorized it with respect to the overall morphology of the larva and the rudiment, and captured images of the whole larva under visible light as well as fluorescent light to assess dye distribution. We excluded any larvae from the analysis that we categorized (blind) as having abnormal gross rudiment morphology. We saw no injection treatment effect on rejected larvae (data not shown), indicating that abnormal rudiment or larval morphology is a non-specific phenotype resulting from our injection protocol in a subset of injected larvae.

Next, we remounted each larva under un-raised cover glass in a very small volume (approximately 20 µl) of MFSW, thus compressing the larva so that all of the individual elements of the juvenile skeleton could be visualized in a much narrower focal plane (see below). Finally, we scored each of these compressed larvae in a single blind fashion (as above), counting and staging all of the visible adult and juvenile spines, including the two right side juvenile spines [40]. We also counted the total number of cross hatches (see below and [28]) in each spine element. While the numbers of visible juvenile spines varied (independent of treatment; data not shown), there were invariably 15 adult spines/spine elements.

## Results

### Morpholino validation

**Figure 1:**
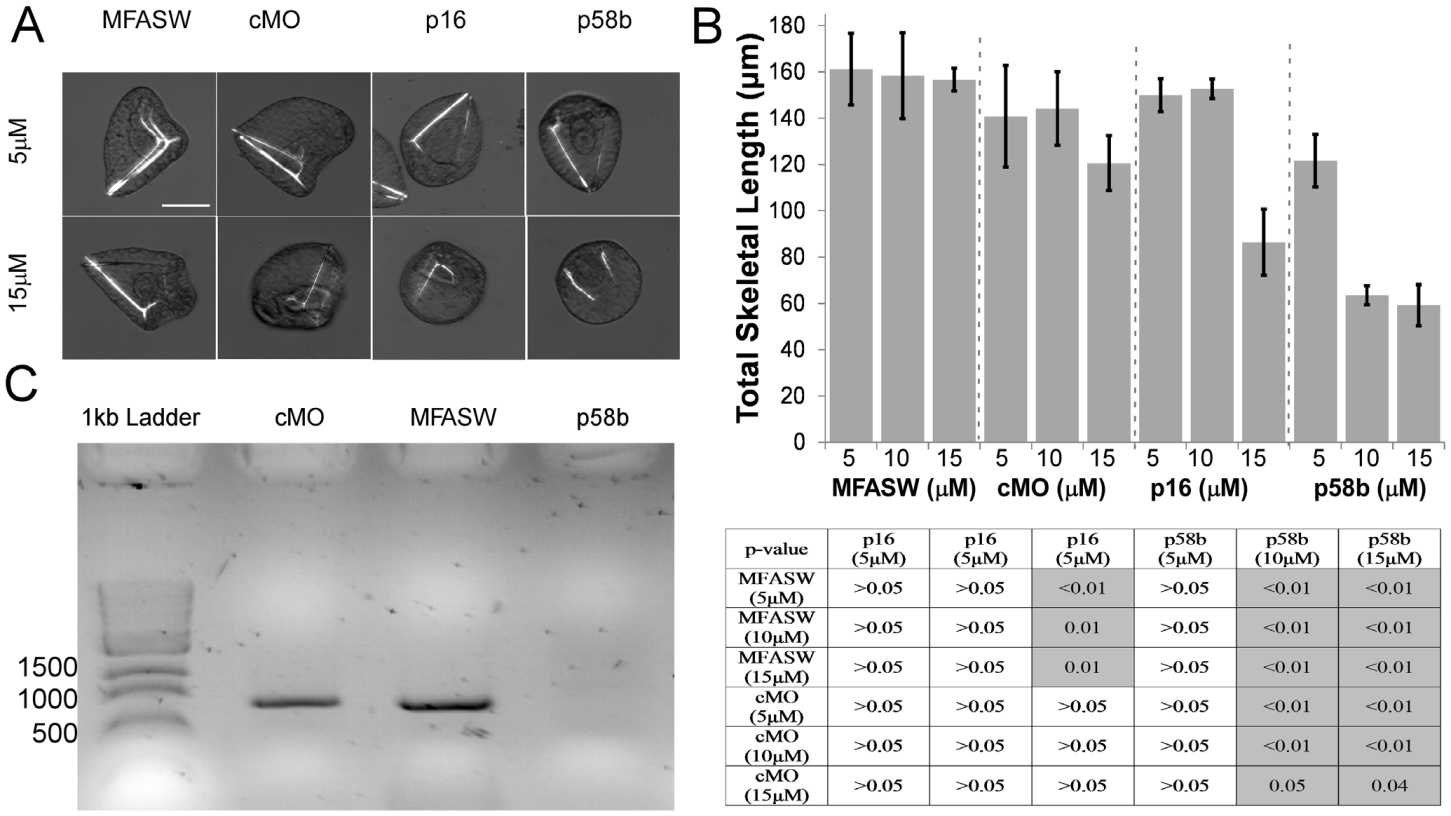
Results from incubation of post-gastrula embryos in p16 and p58b vMOs. **A**) Representative images of embryos exposed to p58b or p16 vMOs for 48 h, compared to control vMO (cMO) and MFASW. **B**) Quantification of embryonic skeletal length for all treatments. Table underneath panel B shows p-values from ANVOVA with post-hoc comparisons using Bonferroni correction. Significant differences (p<0.05) are indicated with darker shading. Note that no significant difference was found between MFASW and cMO at any concentration. Note also that µM MFASW indicates that the same amount of milliQ water was added to MFASW as to the concentrated vMOs. Error bars indicate one standard error of the mean. **C**) RT-PCR of embryos incubated in 15 µM p58b vMO. The visible bands corresponds to the correctly spliced variant of p58b. This variant is absent in the p58b morpholino treatment, indicating knock-down of the correctly spliced variant. Scale bar in A applies to all images and corresponds to 55 µm.

Our p16 and p58b vMOs are identical in sequence to the conventional MOs designed by Cheers and Ettensohn [34] and Adomako-Ankomah and Ettensohn [33], respectively. We validated the vMO formulation of these oligonucleotide sequences by incubating gastrula stage *S. purpuratus* embryos for 48 hours in vMOs and measured skeleton length in prism stage embryos (Fig. 1). Embryos incubated in 15 µM but not 5 µM vMOs for both genes contained shorter and less well-formed skeletal rods than controls (Fig. 1A,B). Embryos treated with vMOs for both genes also possessed significantly shorter PO skeletal rods than sea water controls [70±18 µm shorter for p16, p = 0.01; 97±16 µm shorter for p58b, p<0.01], but this was not true for embryos treated with control vMO (cMO) [36±16 µm shorter, p = 1] – note that all reported values resulted from ANOVA post-hoc comparisons. Therefore, it is clear that, in a high-salt medium (in this case, artificial sea water), these vMOs can cross three plasma membranes and a basal lamina and generate a phenotypic effect. However, there does appear to be a modest growth-delaying effect (Fig. 1B) with the control vMOs, indicating a non-specific effect of morpholinos when used in this manner.

The p58b MO designed by Adomako-Ankomah and Ettensohn [33] is a splice-inhibitor, so in order to further verify the knock-down efficacy of p58b vMO we conducted RT-PCR on embryos treated as above to identify any splice variants. We observed the expected disappearance of the spliced RNA among embryos treated with 15 µM p58b vMO (Fig 1C), but not 5 µM p58b vMO (not shown) or controls (Fig 1C).

### Brief overview of rudiment formation in purple urchins, and the cavities formed therein

**Figure 2:**
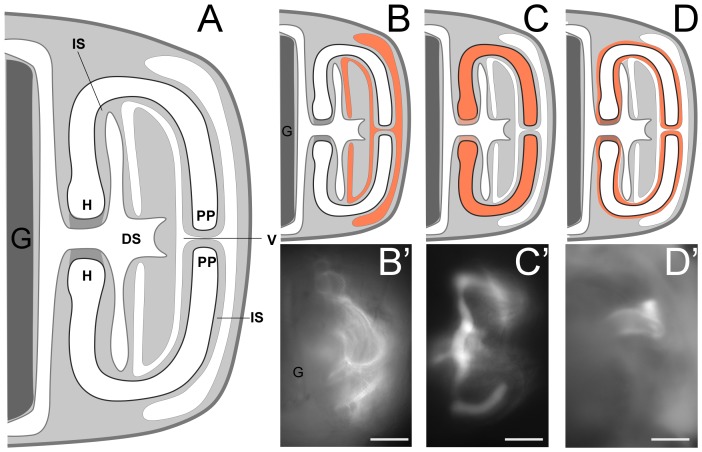
Representative images of rudiments, depicting the most common injection locations. (**A**) A schematic diagram of juvenile rudiment tissue layers. Cavities within the rudiment are shown in white, the blastocoel in light grey, and the stomach in dark grey at left. (B–D) Schematic diagrams of RD fluorescence (orange) in (**B**) the vestibule, (**C**) the hydrocoel (including primary podia) and (**D**) the intercoelomic space. (**B′–D′**) Representative corresponding epi-fluorescent images of RD distribution in these respective regions, one minute after injection. The stomach is in dark grey at left. The area in medium grey in the center of the diagram, to the right of the stomach, connecting the two shown portions of the hydrocoel (H) indicates the out of focus radial canal. All larvae shown and drawn are in anal view sensu [30], posterior down; therefore the "left" side (where the rudiment is found) is seen to the right in these larvae. H =  hydrocoel, V =  vestibule, IS =  intercoelomic space, PP =  primary podium, DS =  dental sac (derived from left somatocoel). IS is contiguous with the blastocoel, but we give it a distinct term for the reasons described in the text. Scale bars in B′–D′ 35 µm.

**Figure 3:**
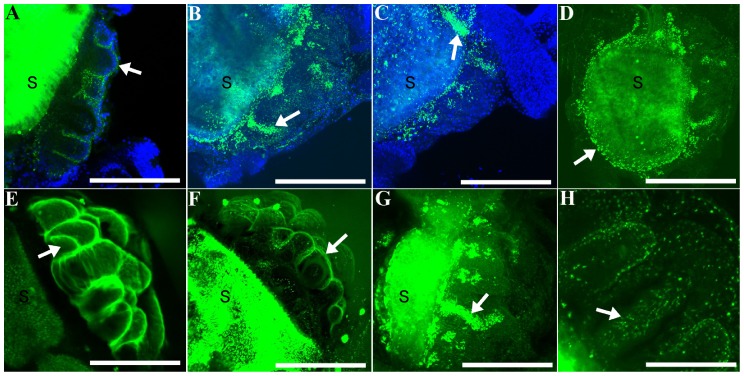
Projections of confocal image stacks, showing the distribution of rhodaminated dextran (RD; green) injected within juvenile rudiments at various time points post-injection (PI), with DAPI (blue in A–C) or with RD alone (D–H). (**A**) Projection of three sections; larva fixed 10 min PI. Arrow points to vestibule labeling (see Fig. 2B): a thin layer of RD between the vestibular ectoderm and the floor of the vestibule surrounding a primary podium. (**B**) Projection of sixteen sections; larva fixed 24 hrs PI. (**C**) Projection of twenty-three sections; larva fixed 24 hrs PI. Arrows in B, C point to labeling in the lumen of primary podia, which is contiguous with the hydrocoel (see Fig. 2C). (**D**) Projection of thirty sections; larva fixed 24 hrs PI. Arrow points to punctate labeling of RD, which has accumulated in the right somatocoel. (**E**) Projection of twenty-four sections; larva fixed 5 min PI. Arrow points to the lumen of a developing spine. Note that in this common "vestibule" pattern, RD labeling surrounds every tube foot and spine element on the oral side of the rudiment. (**F**) Projection of thirteen sections; larva fixed 5 min PI. Arrow points to a thin layer of RD between vestibular ectoderm and the floor of the vestibule surrounding a primary podium; i.e., vestibule pattern. (**G**) Projection of 47 sections; larva fixed 10 min PI. Arrow points to RD accumulation in the lumen of a primary podium. (**H**) Higher magnification projection of nine sections; larva fixed 30 min PI. Arrow points to RD, which appears in a punctate pattern in a spine lumen. Larvae are all oriented approximately as in Figure 2, with the stomach (S) towards the left of each panel, and the rudiment towards the right. Scale bars: A = 100 µm; B = 166 µm; C, D = 133 µm; E−G = 110 µm; H = 63 µm.

The juvenile anlage (or "rudiment") in purple urchin larvae is formed on approximately day 14 (14°C), when a zone of larval ectoderm called the vestibule, invaginates (stage i *sensu* [28]) and then contacts the left hydrocoel (stage ii). Soon thereafter, the hydrocoel adopts the first signs of pentameral symmetry (stage iv), with five primary podia then protruding into the overlying ectoderm (stage v). The juvenile oral-aboral axis is oriented with its oral surface away from the stomach (i.e., towards the vestibule and the left side of the larva). Next, the vestibule itself soon pinches off into an enclosed "vestibular cavity," wrapping tightly around each of the incipient primary podia (*PP* in Figure 2A, below). Further elaborations of the hydrocoel form the spine cavities (see e.g., Figure 3, below), with extensions of the hydrocoel lumen penetrating and connecting each of the forming spines and primary podia (the first five tube feet).

Thus in early stages of rudiment growth and development, there are two distinct cavities: the hydrocoel/tube foot lumen (*H* in Figure 2A, below), which is surrounded by cells of mesodermal origin, and the vestibular cavity (*V* in Figure 2A, below), which is surrounded by cells of ectodermal origin. The floor of the vestibule is the region of vestibular epidermis adjacent to (abutting) the hydrocoel; the roof of the vestibule is the region of vestibular epidermis on the oral side of the juvenile, facing away from the hydrocoel. Later, a third cavity derives from an extension of the fused left and right somatocoels, penetrating the base of the rudiment, and forming the dental sacs (*DS* in Figure 2A, below) among other structures.

### Injection of rhodaminated dextran (RD) to validate injection locations, and the fate of injected compounds

Injection of compounds into a developing sea urchin juvenile rudiment has not been previously reported. Because the injection needle must first pass through the larval epithelium and the vestibular ectoderm before delivery of compounds, a determination of the precise location of the needle tip becomes progressively limited by the optical clarity of the specimen. Moreover, if the desired injection location is the hydrocoel, the needle tip must also pass through the floor of the vestibule as well as the epithelium of a developing primary podium or surrounding epidermis. The decline in the depth of field and numerical aperture associated with higher magnification lenses further reduces the precision with which the injector can discern the location of the needle tip prior to expulsion of compounds from the needle. Therefore, in addition to several preliminary experiments in which we only injected RD, we always co-injected RD with our vMO preparations.

**Figure 4:**
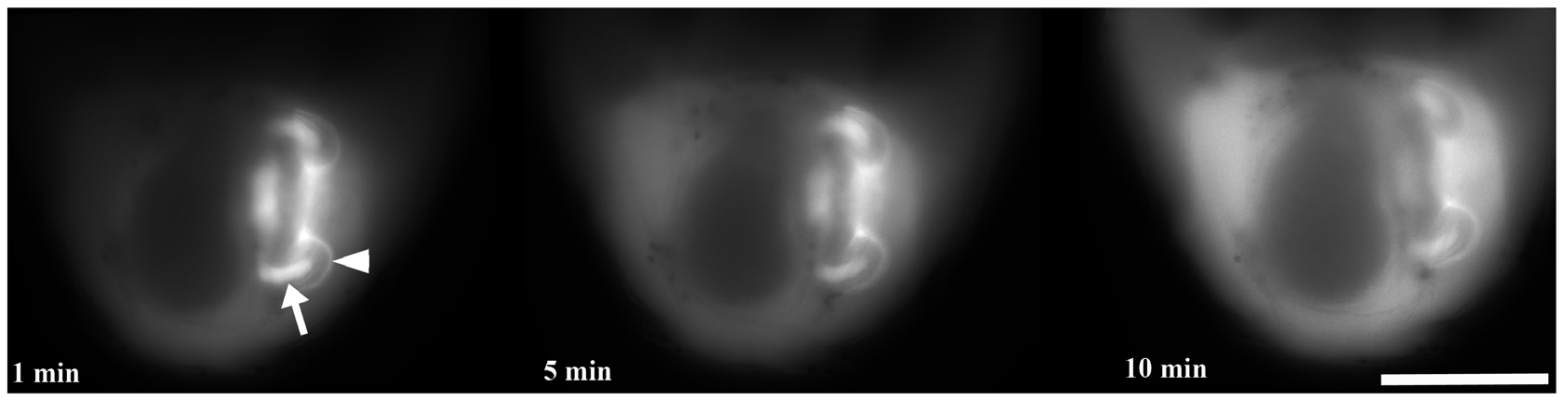
Change in the distribution of rhodaminated dextran (RD) injected into the hydrocoel of a juvenile rudiment during a period of 10 minutes. Note that in addition to strong initial RD accumulation into the lumen of the developing primary podia (white arrow), the RD also labeled the vestibular space in this larva (i.e. the enclosed cavity between the floor and the roof of the vestibule; white arrowhead). Images were not individually adjusted for brightness and contrast and so represent the original relative brightness of RD. The larva imaged was viewed from the anal side *sensu* [30], posterior down; therefore the "left" side (where the rudiment is found) is seen here to the right. The larva imaged was oriented as in Figure 2. Scale bar  = 70 µm.

In a first set of injection trials, we tracked the change in the distribution of RD over a 10 minute (and longer) period, during which some of the RD originally restricted to the juvenile rudiment dissipated into the blastocoel (Fig. 4). Based upon these initial trials, we injected and individually mounted 137 injected larvae on a slide and observed them under epi-fluorescent illumination within 1–2 minutes of injection (Table 2). Owing to variation in the precise site of injection, the initial distribution of RD varied, but the most common pattern was concentration in the vestibule, and less frequently in the hydrocoel or in the space between the two (Figs. 2,4; Table 2), as described further below.

**Table 2:** Summary statistics from several rounds of preliminary injections of rhodaminated dextran into juvenile rudiments.

Trial	# injected	Distribution of dye								
		V	IS	H	B	V+H	IS+H	IS+V	V+H+IS	could not discern
10-Jul	5	0	0	0	1	1	1	0	0	2
12-Jul	24	16	0	3	3	0	0	0	1	1
14-Jul	24	17	0	1	2	2	0	1	0	1
16-Jul	21	11	5	2	2	0	0	0	0	1
17-Jul	24	13	1	1	6	0	0	0	0	3
19-Jul	24	12	0	1	7	0	0	0	0	4
23-Jul	15	8	2	1	2	1	0	0	0	1
total	137	77	8	9	23	4	1	1	1	13
% of total		56.2	5.8	6.6	16.8	2.9	0.7	0.7	0.7	9.5

We assessed the distribution of dye within two minutes after injection. We scored results as the compartment for which staining was brightest. Instances in which more than one compartment was stained with approximately equal intensity were scored as such. In many instances dye leaked into the blastocoel, presumably through the wound from the injection needle. Therefore, if dye was clearly distributed in either vestibule, hydrocoel or intercoelomic space (or combinations thereof) as well as the blastocoel, then we did not record blastocoel staining here. However, we did score blastocoelar staining in instances in which there was no discernible dye in any compartment in addition to staining in the blastocoel. “Could not discern” refers to instances in which we could not make a confident assessment of primary dye location, based upon differential intensity of dye. B =  Blastocoel; H =  hydrocoel (including the lumen of primary podia); IS =  intercoelomic space; V =  vestibule.

Starting at approximately Stage 4 *sensu* [28] the phenotype associated with vestibular injections was a thin area of accumulated RD surrounding each of the primary podia and rudimentary spine cavities across the entire oral side of the rudiment (Fig. 2B; see also Fig. 3E,F). Likewise, hydrocoel injections labeled the entire contiguous hydrocoel space, including the lumen of the primary podia and developing spines (Fig. 2C; see also Fig. 3G). Less common was labeling in the space between the floor of the vestibule and the hydrocoel (*IS* in Fig. 2A, Table 2). Although this space is actually contiguous with the blastocoel (see Fig. 2A), our injections there tended to accumulate RD initially only near the site of injection (Fig. 2D), suggesting that, in the vicinity of the developing primary podia tips, the floor of the vestibule and the hydrocoel are very closely apposed, thus limiting diffusion away from the injection site. For this reason, we believe this rudiment location warrants a special name designation, which we have denoted the "intercoelomic space" (*IS* in Fig. 2A, Table 2). Finally, in those cases where we missed the rudiment completely (approximately 17% of our injections; Table 2), we saw intense RD accumulation throughout the blastocoel immediately after injection.

In a separate set of injections, we stained larvae with DAPI at different times post-injection in order to discern more precisely the distribution of RD in juvenile tissues (Fig. 3). After several hours, RD was typically observed in a punctate pattern (Fig. 3). In most cases (Table 2) RD was detectable in the rudiment, but was also frequently distributed in a narrow zone of tissue that originated from the rudiment, but entirely encircled the gut (Fig. 3B,D,G). We interpret this distribution as labeling of the right and left somatocoels, which encircle the gut at these stages [29],[31],[41]. Fluorescence in the stomach (indicated by an *S* in Figure 3) was not considered to be a reliable indicator of the presence of RD because of algal auto-fluorescence.

Our RD tracing experiments demonstrate that our injections deposited reagents in the vicinity of juvenile skeletogenic cells, and our vMO incubation experiments showed that the vMOs were capable of crossing epithelia and cell membranes in order to reach their target cells. However, because of presumed differences in the behavior of vMOs compared to RD, we could neither infer directly the residence time of the former, nor the efficiency with which they were taken up by target tissues. Note that vMOs with fluorescent tags are not available, as such tags would likely interfere with the ability of vMOs to effectively cross membranes.

### Effect of vMOs on skeletogenesis

**Figure 5:**
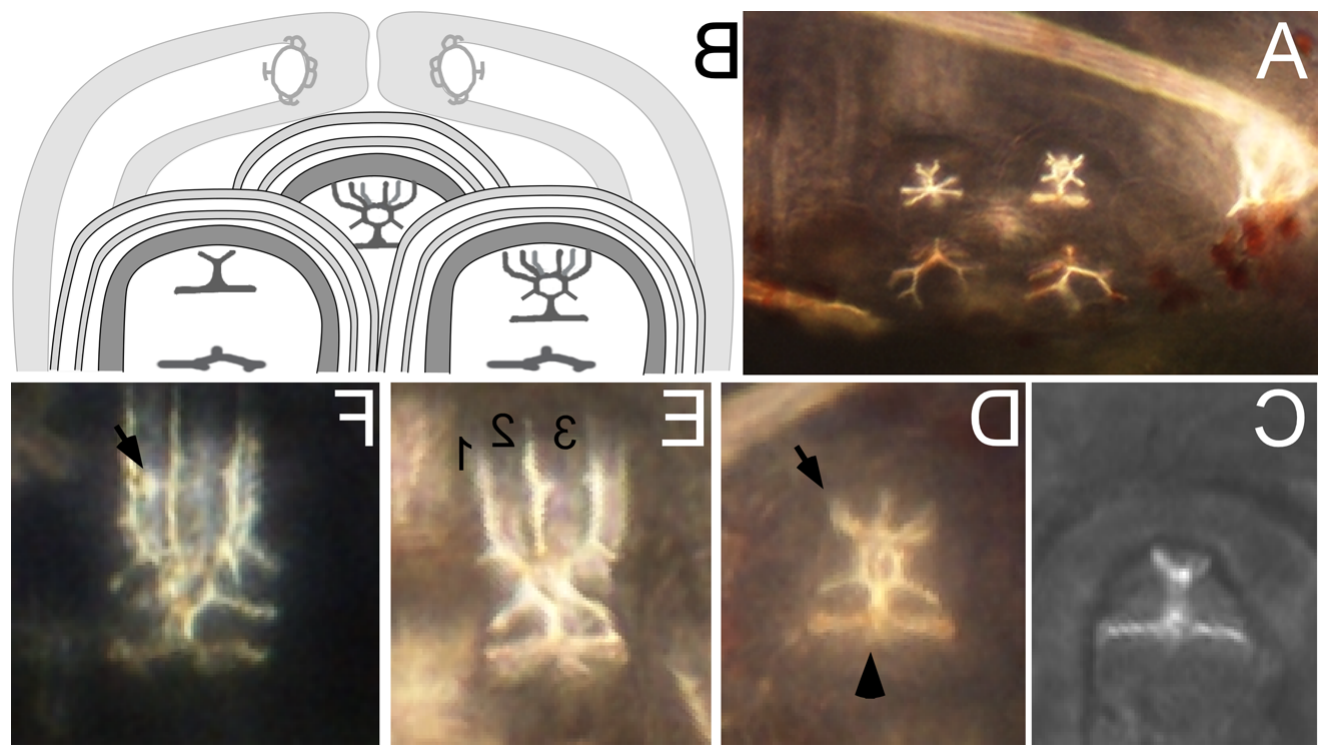
Developing spine structures in *S. purpuratus*. (**A**) Close-up of the rudiment, focused on two of the adult spine cavities and the developing skeletal elements within. (**B**) Cartoon showing the relative arrangement of three adult spine cavities and the surrounding pair of primary podia. (**C–F**) Close-up views of the developing adult spine *anlage* at progressive stages, *sensu* [28]. (**C**) Stage 6 "spine primordium + base". In stage 6 larvae vertical spine fronds are not yet present in any of the 15 adult spine *anlage*. (**D**) Early Stage 7 "pre-spine". Note that six fronds (four or five of which are visible here; arrow) have now started to elongate vertically from the spine base (arrowhead). (**E**) Late Stage 7 "pre-spine". Note that the spine fronds (three of which [numbered] are in focus in this view, the other three are visible but out of focus in the background) have continued to elongate, but no cross bars ("cross hatches") are yet visible. (**F**) Early Stage 8 spine, defined by the presence of at least one complete cross hatch (arrow). In our vMO experiment, we only selected larvae that were at Stage 7; rejecting all Stage 6 and Stage 8 larvae. All images here are from abanal views *sensu* [30], with posterior to the left and the left (rudiment) side up.

**Figure 6:**
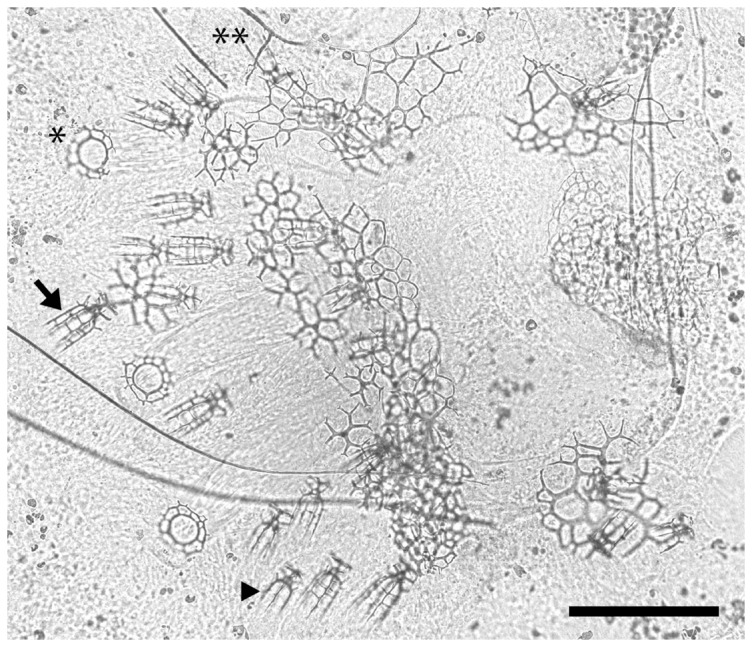
An example of one of our experimental larvae, compressed under cover glass to score skeleton at the end of the experiment (96 hrs after injection – see also **Fig. 5**). Arrow: an adult spine with two cross hatches. Arrowhead: an adult "pre-spine" - so called since it has zero cross hatches. Asterisk: tube foot end plate with two concentric rings. Double asterisk: a juvenile "pre-spine." This larva was injected with p16 vMO, and has under-developed adult spines compared to the control treatments (see Fig. 7). Scale bar  = 89 µm

**Figure 7:**
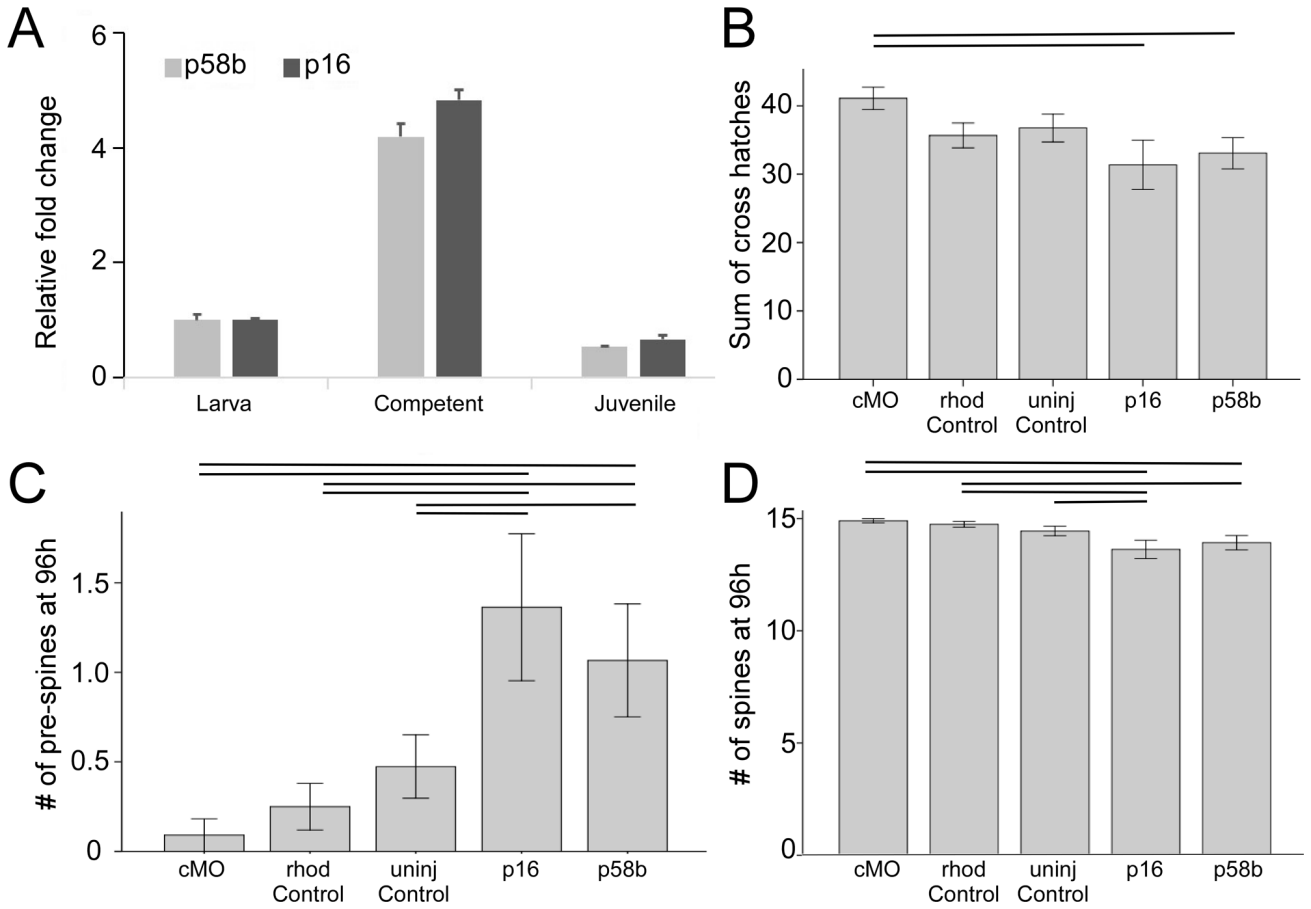
Quantification of vMOs knock-down effect on three aspects of skeleton growth in juvenile rudiments. See Figures 5 and 6 for details on which skeletal elements were scored. A) qRT-PCR results for p58b and p16 in three development stages show expression of these genes in juvenile stages. **B**) spine elongation, measured by the number of cross hatches in adult type spines over a time period 96 h post-injection. **C**) number of adult type pre-spines present in the oral region of the juvenile rudiment 96 h post-injection **D**) number of adult type spines present in the oral region of the juvenile rudiment 96 h post-injection. Lines above bars indicate significant differences (p<0.05) between pairs of treatments.

We injected Stage 7 larvae (Fig. 5) with p16 and p58b vMOs, allowed larvae to develop for up to 96 hours and then scored for differences in juvenile and adult-type [42] spine elongation (Fig. 6). These injections inhibited skeletogenesis of these spine elements in the following ways (Fig. 7; all results below are mean differences ± standard error and p-value from ANOVA post-hoc comparison using simple contrast between all treatments and control vMOs):

- the length of adult spines (as assayed by the sum of the number of cross hatches in the 15 adult spines in each larva; Fig. 6) was reduced in p16 and p58b vMO injections compared to control vMO injections (F_4,67_ = 2.2; p16 = −9.7±3.7 µm, p = 0.01; p58b = −8.0±3.5 µm, p = 0.02; Fig. 7B).
- the number of adult pre-spines per larva (Fig. 7C) was higher in p16 and p58b vMO injections compared to control vMO injections (F_4,67_ = 4.2; p16 = 1.3±0.4, p<0.01; p58b = 1.0±0.4, p = 0.01). This means that we observed more delayed skeletal elements in these treatments. Consequently,
- the number of adult spines per larva from a maximum of 15 (Fig. 7D) was reduced in p16 (1.27±0.4 fewer; p<0.01) and p58b (1.0±0.4 fewer; p = 0.1) (F_4,67_ = 3.4) vMO injections compared to control vMO injections.

## Discussion

Complex life cycles have evolved repeatedly in animals and non-animals alike. In the majority of coastal marine invertebrates, the most dramatic life cycle transition involves a metamorphosis from a planktonic larva to a benthic adult. This metamorphic transition has profound implications for the ecological stability of marine communities, gene flow among them, and their recovery following disturbance. Furthermore, invertebrate metamorphoses are fascinating developmental events in their own right, where the adults often differ morphologically, behaviorally and ecologically from their corresponding larval forms [43]. Echinoderms such as the purple sea urchin, *Strongylocentrotus purpuratus*, represent notable examples.

Despite the relevance and interest in such transformations across invertebrates and within echinoderms, we have limited mechanistic understanding of the developmental and physiological processes that regulate marine invertebrate metamorphoses. One major impediment to gaining such understanding is the difficulty inherent in manipulating genes in late stage larvae without causing embryonic phenotypes at earlier stages; even descriptively visualizing the complex ontogenic events of metamorphosis in live larvae is challenging.

Here we report on our initial successes in overcoming both of these technical challenges. We have developed a technique for injecting reagents into live *S. purpuratus* larvae, specifically targeting the echinus rudiment: a series of tissues that develop within the larval body, and are fated to form predominantly the oral structures of the urchin juvenile. By injecting rhodaminated dextran (RD), we were able to consistently label structures within the rudiment, providing a technique for visualizing these structures in live larvae.

Furthermore, we have manipulated the normal ontogeny of juvenile structures by injecting Vivo-Morpholinos (vMOs) –a class of morpholino oligonucleotides that are designed to cross cell membranes– into various compartments within the rudiment of late stage purple urchin larvae. Specifically, we document an inhibition in growth and elongation of incipient adult spines using vMOs directed against p16 and p58b, two genes known to be involved in skeletal elongation in urchin embryos [33],[34].

We are confident in the specificity of the phenotypes that we report for the vMO rudiment injections for the following reasons: 1) we did not observe skeletogenic phenotypes in controls, and specifically using the control vMO, in either soaked embryos or injected larvae; 2) we were able to provide evidence that p58b vMOs are effectively eliminating the correct splice variant of the gene and consequently lead to a functional knockdown; 3) the phenotypes that we did observe in embryos with our p16 and p58b vMOs (Fig. 1) phenocopy previously published results obtained with standard MOs injected into eggs [33],[34]; and 4) the phenotypes that we observed in our rudiment injections (scored blind) indicated that injected rudiments continued to develop normally after injection, across all treatments – it was only a subset of the skeletal elements specifically in the p16 and p58b vMO treatments that showed inhibited growth (Fig. 7).

Therefore, our results indicate, for the first time, that morphogenesis of the juvenile sea urchin can be manipulated by morpholino injection. As a corollary, we provide evidence that p16 and p58b are required for normal skeletal elongation during sea urchin juvenile skeletal development, as they are during embryogenesis, a finding consistent with the expression of much of the purple urchin larval skeletogenic regulatory network in juvenile rudiments [44].

### The injection technique

Because there is no traceable vMO reagent available at this time, we were unable to directly assess the fate of injected vMO compounds; therefore, we co-injected vMOs with rhodaminated dextran (RD). Whether we injected into the vestibule or the hydrocoel, we were surprised to see a substantial portion of the injected RD leaking out of the rudiment into the blastocoel over the course of fewer than 10 minutes. Furthermore, while some RD labeling was still visible in injected rudiments 24–96 hours after injection, we detected high concentrations of RD labeling in the left and right somatocoel at 24–96 hours. Finally, the dispersion of injected RD changed from homogeneous to punctate. These findings warrant further investigation into the apparently active movement of substances around the various compartments of the larva.

While we cannot use these RD results to determine the fate of our injected vMO reagents, our observations with RD, coupled with our soaking experiments showing the ability of these vMOs to cross purple urchin membranes, as well as our injected vMO phenotypes suggest that our injection technique resulted in accumulation of vMOs in target rudiment tissues. An additional approach would be to use standard (non-Vivo) MOs and mix them with Endo-porter (GeneTools), a substance that facilitates movement of molecules across the plasma membrane.

The basic protocol that we described should be feasible for most inverted microscopes and injection configurations. One of the most significant challenges that we faced was ascertaining the exact location of the tip of the needle relative to the many tissue layers within the echinus rudiment. The injection microscope we used was not equipped with epi-fluorescence; having that capability would have been preferable, as it would have allowed us to confirm injection location without the need to mount the post-injection larvae under raised cover glass. With our set-up, we had the best success when viewing the needle with a partially-closed diaphragm under 200−400x magnification. Watching the liquid being expelled from the needle tip also gave hints as to the location of the injection.

Over the course of over 280 injections that we performed in this study we noticed some variation in injection location; application of this technique requires training to consistently inject MOs into the juvenile rudiment. Still, considering the simplicity of the injection setup as well as the injection itself, it should be relatively unproblematic for any interested researcher to gain experience with this technique.

We also note that different echinoid (and echinoderm) larvae differ in the degree of optical clarity of the rudiment, and how deep within the larval body the rudiment is positioned. Furthermore, the utility of this injection technique outside of echinoderms would be limited in those larvae, for example, that develop within larval shells. Nevertheless, we are confident that our basic injection technique will be broadly applicable among echinoids, echinoderms and representatives of many other phyla as well.

### Tracing rudiment development

Classic histological descriptions of sea urchin rudiment development have depended upon careful and painstaking reconstruction of events from sectioning of fixed larvae at various stages [41],[45]–[50]. Confocal microscopic techniques allow for a more dynamic approach while maintaining the continuity of complex tissue interactions, movements and transformations. By injecting RD into late stage larvae, we have thus obtained what we believe are the clearest three dimensional reconstructions of the multiple tissue layers in the rudiment yet described. Although it is beyond the scope of the current study, we note that 4D descriptions of juvenile ontogeny are possible, which will provide new insights into juvenile morphogenesis in a variety of echinoderms and non-echinoderms alike.

### Manipulating genes in larvae

Metamorphosis has been described as a second embryogenesis [51]: despite fundamental differences in their cellular contexts, both processes are characterized by major morphogenetic events occurring in a relatively short period. Yet for largely technical reasons the 'first embryogenesis' is far better understood. Our hope is that the rudiment injection technique pioneered herein –and its use to deliver morpholinos to presumptive juvenile tissues– will herald a rapid expansion in our understanding of juvenile morphogenesis and the metamorphic transition. Does metamorphosis involve a redeployment of particular embryonic gene networks, and if so, in what way are they modified? In taxa that undergo juvenile morphogenesis within a functioning larval body *sensu* [52], how is that overlap coordinated and how did it evolve? What does it mean -in developmental-physiological terms- for a larva to be competent to undergo its final transformation to the benthos? What is the mechanistic basis for adaptive phenotypic plasticity in larvae? How is diversity in juvenile form generated during ontogeny? Our hope is that the application of gene manipulation techniques across a range of marine invertebrate larvae will help answer these questions, and in so doing highlight metamorphosis as a fruitful paradigm for interdisciplinary studies into ecology, evolution, physiology and development.
