# Correction: Manipulation of Developing Juvenile Structures in Purple Sea Urchins (*Strongylocentrotus purpuratus*) by Morpholino Injection into Late Stage Larvae

**DOI:** 10.1371/journal.pone.0117679

**Published:** 2015-01-27

**Authors:** The PLOS ONE Staff

There is an error in Fig. 5, “Developing spine structures in *S. purpuratus*.” Please see the corrected Fig. 5 here.

**Figure 5:**
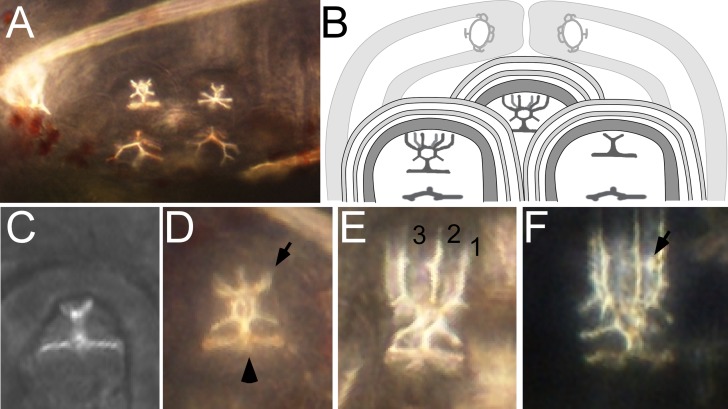
Developing spine structures in *S. purpuratus*. (A) Close-up of the rudiment, focused on two of the adult spine cavities and the developing skeletal elements within. (B) Cartoon showing the relative arrangement of three adult spine cavities and the surrounding pair of primary podia. (C–F) Close-up views of the developing adult spine *anlage* at progressive stages, *sensu* [28]. (C) Stage 6 “spine primordium + base”. In stage 6 larvae vertical spine fronds are not yet present in any of the 15 adult spine *anlage*. (D) Early Stage 7 “pre-spine”. Note that six fronds (four or five of which are visible here; arrow) have now started to elongate vertically from the spine base (arrowhead). (E) Late Stage 7 “pre-spine”. Note that the spine fronds (three of which [numbered] are in focus in this view, the other three are visible but out of focus in the background) have continued to elongate, but no cross bars (“cross hatches”) are yet visible. (F) Early Stage 8 spine, defined by the presence of at least one complete cross hatch (arrow). In our vMO experiment, we only selected larvae that were at Stage 7; rejecting all Stage 6 and Stage 8 larvae. All images here are from abanal views *sensu* [30], with posterior to the left and the left (rudiment) side up.
